# Evidence Is Evidence: An Interview with Mary-Claire King

**DOI:** 10.1371/journal.pgen.1003828

**Published:** 2013-09-26

**Authors:** Jane Gitschier

**Affiliations:** Departments of Medicine and Pediatrics and Institute for Human Genetics, University of California, San Francisco, San Francisco, California, United States of America

I often have one eye on the *PBS NewsHour* when I'm preparing dinner, but whenever the subject turns to the Supreme Court, I snap to full attention. I love the *NewsHour*'s Marcia Coyle who, like her equally engaging counterpart Nina Totenberg on National Public Radio, bears witness to the Court's exertions and delights in sharing them with us. In an age of 24/7 tweets and Snapchats, courtroom drawings and the recollected volley of question and answer are seeming throwbacks to the origins of the Constitution itself. We imagine ourselves there, we know this really matters, and we wonder what the Court will decide.

My American colleagues in human genetics and I are now following one Supreme Court case in particular: Association for Molecular Pathology v. Myriad Genetics, which is under consideration as I write this. The plaintiffs charge that patents held by Myriad on the “breast cancer genes” *BRCA1* and *BRCA2* are invalid and unconstitutional; the Court's ruling will likely have broad implications for the patenting of human genes and the diagnosis of inherited human diseases. It seems a fitting moment to reflect on the saga of Mary-Claire King (Image 1), whose tenacious work underlay the discovery of *BRCA1*. Now at the University of Washington, King had just completed her graduate work when she was offered a research post at UCSF (University of California, San Francisco) by Nicholas Petrakis to address a genetic role for breast cancer. King, later setting up her own laboratory at the University of California, Berkeley, designed studies involving large-scale recruitment of families, explored mathematical models to explain the data gathered from them, and, after a 17-year heroic effort, discovered the *BRCA1* locus on Chromosome 17 by linkage in families with early-onset breast cancer.

**Figure:**
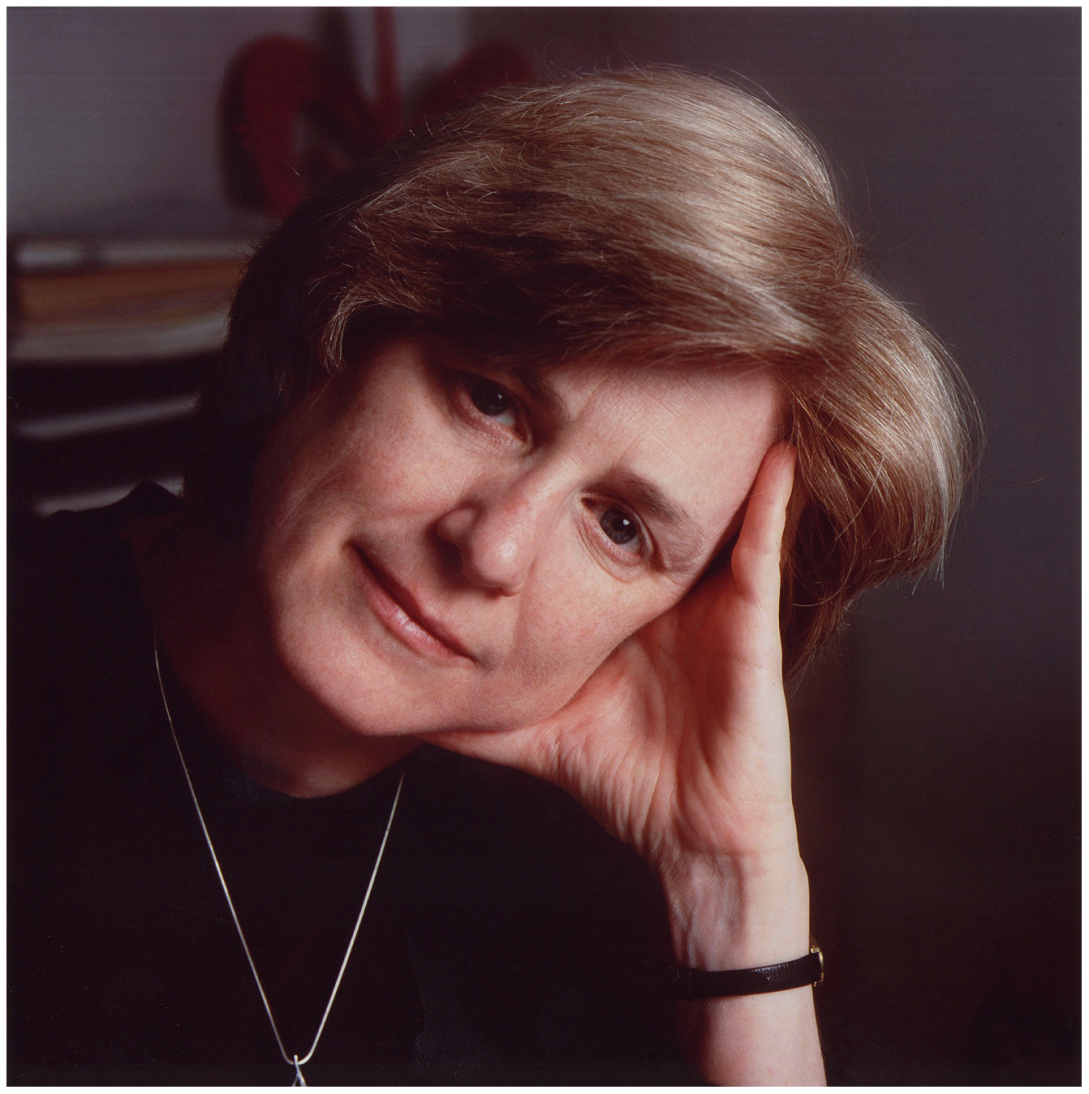
Mary-Claire King. Photo courtesy of Mary-Claire King.

Yet, discovery of the *BRCA1* locus wasn't King's only, or even her first, major contribution. Her graduate work with Allan Wilson made the cover of *Science* magazine, as it employed protein polymorphisms to sharply narrow the genetic distance between humans and chimpanzees. Over the course of her life, King became involved in a wide range of projects, including applications of genetics to human rights, most notably among the disappeared grandchildren of Argentina, and projects that made inroads into genetic underpinnings for deafness and for schizophrenia. Still, for this interview, I chose to focus on *BRCA1*, which figures so prominently in our culture that a new movie on it has been produced (with King portrayed by Helen Hunt). During a quick Mother's Day visit to my daughter Annie, now a freshman at the University of Washington, I convened with King on a warm Saturday morning in her campus office, its shelves still bulging with white binders filled with pedigrees of families with breast cancer.

**Gitschier:** When I looked back over your career, I was impressed with a kind of fearlessness that seemed to recur in terms of initiating new projects. Do you feel that you always had a lot of self-confidence? I'm wondering if somehow Allan Wilson, who was an early mentor and who is clearly very adventurous…

**King:** …clearly fearless.

**Gitschier:** Did he help you to gain the confidence to go after big problems, big questions, and not be intimidated?

**King:** Thanks very much for saying that you consider the work a reflection of fearlessness; that's probably the nicest thing that anybody has ever said about the work.

I think the adventurousness is true, but it's *not* self-confidence. I think it's because I can't resist doing interesting things. It's like the little tag line on your e-mail [from poet Mary Oliver: “Tell me, what is it you plan to do with your one wild and precious life?”]: basically, you only live once. Otherwise you'll forever feel terrible that you didn't go for the ring, for a way of making a major impact on a major area.

Allan had a huge effect on me in many ways: to believe in evidence and to be very methodical and very self-critical, and then when you are *sure* about the data, unless somebody can literally disprove you with better data, to not be intimidated by *anybody*. He never said any of that explicitly, but that was the culture of his lab and it was the culture of his life.

You did not grow up in the Wilson lab thinking that you were right! We were extremely critical of ourselves and of each other. Persistence was also part of the culture of the Wilson lab and it is part of me. It took 17 years to map *BRCA1*!

Being comfortable with uncertainty *for years* was the third lesson of the Wilson lab, and it is an essential part of what we do.

**Gitschier:** That's a Buddhist principle. In fact there is a book of that title [*Comfortable with Uncertainty* by Pema Chödrön].

**King:** Ah, that's interesting; Allan would have approved.

**Gitschier:** So you leave Allan's lab with a sensational story in *Science*, and now you move to work on breast cancer with Nick Petrakis. How did you hit it off with him?

**King:** Oh, extremely well! He's just the loveliest person and gave me complete freedom to run with what I wanted to think about. I knew nothing about breast cancer, but to me it meant that the same kind of approach that one uses to look at long-term evolutionary processes could be used to look at extremely short-term evolutionary processes, both within a family, where one could track susceptibility, and in a tumor, where a tumor is constantly subject to mutation, selection, and migration.

In fact, getting back to your original question, one ingredient for success is paradoxically quite different from what you suggested. When women our age started in the field, there were very few of us, and we were absolutely on the margins. People pretty much ignored us. I have come to realize that there was a great freedom in being ignored, that you could go after huge questions, because nobody noticed.

So, when I started working on breast cancer with Nick, I would ask for help, primarily from surgeons because they knew about the phenotype and they knew their patients. They were older, they were without exception male, and they were wonderful to me. I was obviously no threat.

**Gitschier:** Did you two have a specific plan?

**King:** I thought, if this is going to work, we're going to have to prove that there is such a genetic effect. There was very nice epidemiologic data going back to the 1920s from English statistical surveys, by a remarkable physician-scientist named Jane Lane-Claypon, one of the founders of modern epidemiology. Dr. Lane-Claypon showed that among women whose mothers had died of breast cancer, breast cancer was two- to three-fold more likely to cause their death than among women whose mothers had died of something else. It was also true for cancers like lung, largely because of exposure to carcinogens in mining districts. There wasn't a hypothesis about *why* there was familial clustering; the nature of it and whether it would fit a genetic model were up in the air.

So I tried to do two things at once, which proved to be a good idea. One was that I tried to get good data that I could use to create mathematical models and then test them. We piggybacked onto an NCI [National Cancer Institute] study that was being done for a totally different reason—the question of whether birth control pills altered risks of breast, ovarian, or uterine cancer. This was the 1970s and a very large number of women were being interviewed for a detailed history of the use of oral contraceptives.

I remember saying, “Would it be all right if I added a few questions about family history?” and the NCI staff, I think thanks to Nick's introductions, thought, “Well, what kind of damage can one girl possibly do?” It wasn't too long before the family history questions threatened to overwhelm the project! The interviewers were fabulous, and we ended up with 1,500 pedigrees based on reports of cases and another 1,500 based on reports of controls.

**Gitschier:** At this point you had started your own lab at UC Berkeley.

**King:** Around then. As a postdoc, I was still living in Berkeley, and for months I took my infant daughter Emily to UCSF with me, and I had someone care for her there, because I was breastfeeding. That was not a long-term solution. I saw an ad in *Science* for a tenure-track assistant professorship in the Division of Epidemiology in the School of Public Health [at University of California, Berkeley]. So I thought, epidemiology is really population genetics and evolutionary principles applied to disease distributions. I can do that.

It's actually a very interesting story about how I got the job.

**Gitschier:** Well, let's hear it!

**King:** This was just when all faculty positions in all public institutions nationally had to be advertised, though the Division had a person in mind for this job. It was also the beginning of affirmative action, which meant that if a woman or a member of a minority applied for a position, the person either had to be interviewed or an explicit statement had to be made about why that person was not interviewed. I learned later that it was perceived by the people on the committee to be easier to interview me than to write this statement, that surely I would be an unqualified nonspecialist when they interviewed me.

My job seminar was in a small classroom, but at least 200 people tried to get in. The head of the search committee looked all around and said, “Who are these people?” This was 1975, just after the *Science* paper had come out, and these were people from the Wilson lab and from other Berkeley labs. I said, “These are people who are interested in how genetics can contribute to epidemiology.” And he said, “There is more to this story than that!” Weeks passed and then I got a call offering me the job. I was dumbfounded and delighted. And *then* I found out what had happened.

One of the other reforms was that search committees had to have a woman or minority member, or both, and also had to have a student member, or explain why not. Their student member was also their woman member, and her name is Cathy Schaefer.

**Gitschier:** Oh! Cathy is now co-head of the big Kaiser [Permanente] genetic epidemiology project.

**King:** Cathy is responsible for my career. We did not know each other at that time, of course. She was apparently a bulldog, on the principle that this small division needed to have additional disciplines, to include genetics, and to have a woman as an assistant professor. According to the head of the committee, “She just got her teeth into it and wouldn't let go.”

I am absolutely a child of affirmative action. After I had accepted the job, the division head said to me, “I just want you to know that you are only here because of all these new regulations, and we are really scraping the bottom of the barrel in hiring you.” And I said, “We'll see how long you feel that way!”

**Gitschier:** OK, let's return to these two parallel approaches: first, to the interview data and its analysis. I understand that the *PNAS* [*Proceedings of the National Academy of Sciences of the United States of America*] paper that resulted much later [in 1988], with Beth Newman as first author, is one of your favorite papers.

**King:** Yeah, it is!

**Gitschier:** Why is that?

**King:** Because it is purely mathematical. It's the use of multivariate, complex segregation analysis in a way that had never been used before. Complex segregation analysis was intended to be used for genetic traits in order to understand whether the trait is dominant, recessive, fully penetrant, and so on. It was developed by mathematical geneticists Newton Morton and Robert Elston.

We didn't try to do anything untoward with the mathematics, but our underlying idea was to ask the question: *is* there an inherited form of breast cancer? Can we, out of the 1,500 families, state genetic hypotheses and determine likelihoods of those genetic hypotheses based on the distribution of breast cancer in the families, each ascertained through one breast cancer patient? Then can we test statistically whether those genetic hypotheses are better fits to these data than are other hypotheses of clustering in the absence of a genetic effect? And if so, can we predict the parameters of the genetic hypotheses?

These heroic interviewers had asked every index case and every control (age-matched from the same neighborhoods) about the history of cancer in their sisters, mother, maternal and paternal aunts, and maternal and paternal grandmothers. Unfortunately, we found immediately that people knew the lineages of their mothers *much* better than they knew the lineages of their fathers. So we had to limit all our analysis to mothers and sisters; we weren't able to use all that lovely data on aunts and grandmothers because it was so skewed to knowing about mothers. But with 1,500 families we *still* had enough data to address the questions.

We set up all possible hypotheses and tested them. The best model *by far* was an autosomal dominant model that explained 4%–5% of the cases in this cohort. It predicted a “lifetime” risk of breast cancer among carriers of 85%, which proves to be spot on. A nice *p*-value is not biological proof, but it did give me a sense that we were on the right track.

We were slow to publish the statistical work because I had this illusion that I would put the statistics together with the molecular genetics in a single paper once we found linkage. But Luca Cavalli-Sforza, who sent this paper to *PNAS* for us, said, “Don't wait. Put this out now because you're going to need funding to scale up.”

**Gitschier:** Good point! So now let's talk about the second approach, which led to linkage to *BRCA1* and the big *Science* paper in 1990, with Jeff Hall as first author.

**King:** In parallel to this mathematical project, we were ascertaining families with multiple cases of breast cancer and trying to see if we could identify markers on chromosomes to track cancer through the family. We began years before the era of DNA markers, then incorporated DNA markers as technology improved.

**Gitschier:** Exactly. HLA, blood groups, cytogenetic markers.

**King:** There were many protein markers. I had just done a project with 64 different enzyme assays for the human–chimpanzee project. In fact, the first linkage papers in cancer—not mine—are with esterase D and retinoblastoma. So it was a matter of chipping away at it.

Then, cast your mind back to when RFLPs [restriction fragment length polymorphisms] first became available in the early ’80s and the CEPH [Centre d'Étude du Polymorphisme Humain] project began; we were trying to map markers onto pedigrees. This was pre-PCR, just genotyping markers on Southerns [blots] using DNA from blood. That meant that in order to be able to map a trait, you had to be able to take blood from the person that you were going to genotype. When you are dealing with a fatal disease, that's a terrific problem. If the woman with breast or ovarian cancer was no longer alive, we needed a situation where she had four or more surviving adult children who were willing to give us blood so we could reconstruct her genotype. And we could do that if the marker is variable enough.

**Gitschier:** Oh I see, and then you ask which of the mother's reconstructed genotypes goes with her breast cancer. This is a huge problem!

**King:** This *is* a huge problem. So how did we find families? I owe this to Nancy Reagan, strangely enough.

In 1987, the NCI had a birthday and wanted to highlight projects of various sorts scattered around the country. My little breast cancer project was one of these, and a local television reporter came over from San Francisco and interviewed me. It became obvious to him immediately that we needed more families to be aware of the project, and I said it would be fabulous to use this opportunity to get the word out, which we did. The interview showed that evening on local TV. The next morning, President Reagan announced that Mrs. Reagan had breast cancer. So, the national [television] network then asked what they might have recently archived about breast cancer. My little interview tape was archived in Los Angeles, and was shown on something like 125 affiliates around the country that evening. We were inundated with people contacting us.

We sent out a staff person to enroll women and draw blood from families all over the country. Of course, airport security wasn't what it is now; it was much easier. We could get on a plane, change our reservation at the last minute, and take all the blood samples on board with us.

Meanwhile, we were continuing to use every available genetic marker and to convert markers to PCR once that became available in 1986. Also, all this time we were working with CEPH to place the markers on the genetic map.

**Gitschier:** Let's talk about the insight of looking at age dependency on linkage.

**King:** We were working with 23 large families and genotyping 173 markers, each painstakingly. We had a hodgepodge of results. We had some individual families that were clearly convincing for linkage of breast cancer to a VNTR [variable number tandem repeat] marker on [Chromosome] 17q, and then many that were not. Of course everything was done by hand because none of the analysis was computer-based. We literally had the pedigrees rolled out across lab benches and floors. Then Beth Newman had the idea of arranging the pedigrees in the hallway in order by average age of breast cancer diagnosis in that family.

**Gitschier:** Why did she do that?

**King:** The results of epidemiologic studies from other groups had shown that women who were diagnosed with breast cancer young [i.e., premenopausal age] were more likely to be in families with multiple cases than were women who were diagnosed older. So Beth said, “Let's just look at this by age.”

For each family we were calculating a LOD [logarithm of odds] score—odds in favor of or against linkage—and we realized that as we moved from the youngest-onset families to the older-onset families, that the LOD scores [on Chromosome 17q], with the exception of one family that turned out to have a recombination between our favorite marker and *BRCA1*, were consistently positive up until we got to a median age of about 51.

**Gitschier:** Did you already have a decent LOD score for this marker without factoring in the age? Because you couldn't have done this with all 173 markers.

**King:** Oh, of course we did! There are statistical tests you can make of the significance of your LOD score as well as of the heterogeneity of your data. For these data we had extremely good evidence for heterogeneity, even without taking age into account. Not only did we have a few families that looked extremely promising, but the markers on 17q were the only ones with a very strong *p*-value for heterogeneity. We didn't know the basis for the heterogeneity and we didn't care. We just wanted to find evidence for a first gene.

When we stratified by age, the youngest families had the strongest evidence [for linkage], the middle families had mixed evidence, and the oldest families had negative evidence. In retrospect, among the middle and older families, some were *BRCA2* families, and some we still haven't solved.

**Gitschier:** So when you then got this result, what was the sentiment of the lab?

**King:** That we thought we had something. Evidence is evidence. The result became clear to us in September 1990, and I presented the story in October at the ASHG [American Society of Human Genetics] meeting in Cincinnati. Louise Strong did me the huge favor of adding me to an invited cancer session, with a talk at 10:30 at night. Not that many people came because it was also the first night of the World Series, which was also in Cincinnati.

I presented our data as an interesting story. I knew it was statistically robust, but I was concerned it might be some elaborate fluke. Several groups, including those of Mark Skolnick and Gilbert Lenoir, who had been at the talk, asked for the markers and primers, so we sent all those out. In those days, there was still universal sharing of markers and primer sequences.

A couple of months after the ASHG meeting, Walter Bodmer and Ellen Solomon organized a meeting in England to discuss breast cancer genetics. By then our paper had appeared in *Science*. I don't remember anyone from Utah [i.e., Skolnick's group] at the London meeting, but Gilbert Lenoir from Lyon was there and greeted me warmly.

So I gave my talk, and then Gilbert spoke. He presented what I interpreted as a summary of my talk, with extremely familiar results, assuming that he would go on to describe his results next. But he stopped. I asked what were *his* results, and he said, “Those *are* mine!” His results were virtually identical to ours! Same markers, same age effect, same lovely fit to the same model, exactly our results but on unrelated families. Also, his collaborator Henry Lynch from Omaha had selected families with both breast *and* ovarian cancer, so Gilbert was able to evaluate families with both cancers. *That* was when I believed the result was real.

**Gitschier:** Once you have linkage, the race to the gene is really on, and everybody gets to regroup and leave the gate at the same time!

**King:** That's right. I think it's *very* difficult for people who didn't grow up in our era to understand this, because now we just go to the genome sequence! This was the *reason* for the genome project, so that it wouldn't take four years and more than a hundred people to identify a gene. Now the gene discovery would be a couple of months. But in the early 1990s, you had to *create* the genome sequence as you went along.

**Gitschier:** Absolutely.

**King:** And once there was competition, there was no more sharing of markers and families, other than with collaborators. There were multiple groups working on the search.

**Gitschier:** Myriad [Genetics] was one of your main competitors, and I wonder if you feel comfortable talking about Myriad during this time period.

**King:** I'm most comfortable saying what Maynard [Olson] said, at about the time it ended.

**Gitschier:** You mean…

**King:** When Myriad announced partial sequence. That given the amount of work from scratch that every group had to do, it was a matter of capacity, and they had a hundred times the capacity that we had. It was a combination of luck, driven by your capacity to sequence. We were inside the gene and didn't know it. Everybody had the same strategy, which was to close in as far as possible with linkage—in retrospect, we closed in to a distance of just under a megabase by linkage—and then, to take YACs [yeast artificial chromosomes], cosmids, BACs [bacterial artificial chromosomes] to span the region, then to probe cDNAs from a critical tissue. Then sequence small segments in the region in DNA of affected individuals from different families. You can imagine; well, you know!

**Gitschier:** I do!

**King:** It is extremely tedious. Also, we did this all by hand, but Myriad had an ABI [Applied Biosystems] machine, which made their work a lot faster. I think it would have been a few to several weeks and we would have sequenced our way into exons, so it was very close.

Myriad published the amino acid sequence before they published the DNA sequence. Harold Varmus had just become the director of NIH [the National Institutes of Health] and insisted that they publish what cDNA sequence they had. Their original sequence had an Alu as an exon, and there were a few other smaller errors. We sequenced across the exons in our families, corrected the errors, and immediately found ten different mutations responsible for the disease in ten families.

There was about a three-week gap between their publication of the cDNA sequence and the 1994 ASHG meeting, and Hunt Willard, head of the program committee, let me give a talk—a confirmation talk—in the late-breaking session, which became a breast cancer session.

**Gitschier:** This Myriad patent dispute must have some personal valence for you. I'm just wondering whether you filed an amicus [curiae] brief, for example, when the original suit was filed?

**King:** I'm happy to talk about it now because the Supreme Court has heard it. My view on the lawsuit was that the best thing was to stay personally out of it. The patent monopoly is a problem for women with breast cancer, their family members, women at risk for breast cancer, physicians, advocacy groups. The patent monopoly is not my story.

**Gitschier:** It does have broad implications though for any genetic testing.

**King:** Absolutely! But I think it's important that this not look like sour grapes on my part. So I've not been a plaintiff. Of course, I've been kept informed, and when I've been asked specific questions, I've given specific answers about chronology and problems with the Myriad sequence, and consequences of those problems. I have not been hesitant to say what I know to be facts. ASHG filed an amicus brief, but I actually recused myself, because I was president [of ASHG] when it was filed.

I think there is one issue that many geneticists don't appreciate, which was germane during this whole period, and that was that it's not really the patent per se that is the problem: it's the exclusivity of the licensing of the patent. I suspect that it did not occur to anybody at the time what the consequences would be of a close-to-full-length sequence being patented by a company that would then aggressively enforce a monopoly on it. I certainly didn't appreciate it.

**Gitschier:** Before we close, I must ask you about the new movie, *Decoding Annie Parker*. I understand that you have now seen it.

**King:** I have seen the movie. I did not know about it in advance. I was not consulted or even informed, and I was concerned. A friend of mine who is a movie producer said that there was no good reason not to have consulted me if the idea was to make an honest movie. So I worried, but I honestly didn't take the time to take any formal action. I thought, if I try to actually take this to court, it's going to take forever and life is too short.

**Gitschier:** What was your concern?

**King:** My major concern was that it would be alarmist. Now I've seen it and, thank God, it's not alarmist.

**Gitschier:** What did you think about Helen Hunt portraying you?

**King:** Well, Helen Hunt is a really good actress. She's terrific. I only wish I had the grace of Helen Hunt!

**Gitschier:** But it must seem weird to see yourself on film in a different person.

**King:** It doesn't feel like me. The character has my name but is simply a character. As it turned out, there are very few Helen Hunt scenes. The director subsequently told me that he had removed almost all of the science scenes because focus groups did not like them. Seeing the ones that remain, I can see why that was true. They are dull. The director said, “It was so turgid to describe the science,” and I thought to myself, “I can describe science in a gripping way in three languages! Why didn't he ask me?” We could have given him script that would have been engaging, informative, not turgid, and his focus groups would have loved the scenes.

**Gitschier:** Are you going to be invited to the big opening?

**King:** Of course! The director is distributing the film through advocacy groups and the American Cancer Society. It will be shown at the Seattle International Film Festival as a benefit for SIFF [Seattle International Film Festival] and for us! Overall the film is very positive. It is the story of a young woman's maturing from a flibbertigibbet to a person who is very serious, very focused, uses her intelligence very effectively, and finds out what is going on. It is totally her story.

The acting in the film is consistently excellent! I think for its target audience it will do well. It's a girl empowerment film and it makes that point very well.

**Gitschier:** So, overall a thumbs up?

**King:** It has certainly turned out to be OK. Movies are movies.

*Postscript: A few days after our interview, Academy Award–winning actress Angelina *J*olie announced in an op-ed piece in *The New York Times* that she has a *BRCA1* mutation and underwent a prophylactic double mastectomy, generating further awareness for breast cancer health as well as further discussion about options. Samantha Morton won the Best Actress Award of the SIFF for her portrayal of Annie Parker. And on *J*une 13, 2013, in the case of Association for Molecular Pathology v. Myriad Genetics, the Supreme Court unanimously ruled that human genes may not be patented.*

